# Loss of the BMP Antagonist, SMOC-1, Causes Ophthalmo-Acromelic (Waardenburg Anophthalmia) Syndrome in Humans and Mice

**DOI:** 10.1371/journal.pgen.1002114

**Published:** 2011-07-07

**Authors:** Joe Rainger, Ellen van Beusekom, Jacqueline K. Ramsay, Lisa McKie, Lihadh Al-Gazali, Rosanna Pallotta, Anita Saponari, Peter Branney, Malcolm Fisher, Harris Morrison, Louise Bicknell, Philippe Gautier, Paul Perry, Kishan Sokhi, David Sexton, Tanya M. Bardakjian, Adele S. Schneider, Nursel Elcioglu, Ferda Ozkinay, Rainer Koenig, Andre Mégarbané, C. Nur Semerci, Ayesha Khan, Saemah Zafar, Raoul Hennekam, Sérgio B. Sousa, Lina Ramos, Livia Garavelli, Andrea Superti Furga, Anita Wischmeijer, Ian J. Jackson, Gabriele Gillessen-Kaesbach, Han G. Brunner, Dagmar Wieczorek, Hans van Bokhoven, David R. FitzPatrick

**Affiliations:** 1 Medical Research Council Human Genetics Unit, Institute of Genetics and Molecular Medicine, Western General Hospital, Edinburgh, United Kingdom; 2 Department of Human Genetics, Institute for Genetic and Metabolic Disorders and Nijmegen Centre for Molecular Life Sciences, Radboud University Nijmegen Medical Centre, Nijmegen, The Netherlands; 3 Departments of Paediatrics, Faculty of Medicine and Health Sciences, United Arab Emirates University, Al-Ain, United Arab Emirates; 4 Regional Service for Diagnosis, Prevention, and Care of Birth Defects, Department of Medicine and Aging Sciences, Section of Preventive and Social Pediatrics, G. D'Annunzio University, Chieti, Italy; 5 Department of Orthopaedics and Trauma, University of Edinburgh, Royal Infirmary of Edinburgh, Little France, Edinburgh, United Kingdom; 6 Division of Genetics, Department of Pediatrics, Albert Einstein Medical Center, Philadelphia, Pennsylvania, United States of America; 7 Department of Pediatric Genetics, Marmara University Hospital, Istanbul, Turkey; 8 Ege University, Medical Faculty, Department of Pediatrics, Izmir, Turkey; 9 Institut für Humangenetik der Johann Wolfgang Goethe Universität, Frankfurt, Germany; 10 Unité de Génétique Médicale, Faculté de Médecine, Université Saint Joseph, Beirut, Lebanon; 11 Department of Medical Genetics, School of Medicine, Pamukkale University, Denizli, Turkey; 12 Al-Shifa Trust Eye Hospital, Rawalpindi, Pakistan; 13 Department of Pediatrics and Department of Translational Genetics, Academic Medical Center, University of Amsterdam, Amsterdam, The Netherlands; 14 Serviço Genética Médica, Hospital Pediátrico de Coimbra, Portugal; 15 Department of Clinical Genetics, S. Maria Nuova Hospital, Reggio Emilia, Italy; 16 Department of Pediatrics, University of Lausanne, Switzerland; 17 Institut für Humangenetik, Universität zu Lübeck, Lübeck, Germany; 18 Institut für Humangenetik, Universitätsklinikum Essen, Essen, Germany; University of Oxford, United Kingdom

## Abstract

Ophthalmo-acromelic syndrome (OAS), also known as Waardenburg Anophthalmia syndrome, is defined by the combination of eye malformations, most commonly bilateral anophthalmia, with post-axial oligosyndactyly. Homozygosity mapping and subsequent targeted mutation analysis of a locus on 14q24.2 identified homozygous mutations in *SMOC1* (*SPARC-related modular calcium binding 1*) in eight unrelated families. Four of these mutations are nonsense, two frame-shift, and two missense. The missense mutations are both in the second Thyroglobulin Type-1 (Tg1) domain of the protein. The orthologous gene in the mouse, *Smoc1*, shows site- and stage-specific expression during eye, limb, craniofacial, and somite development. We also report a targeted pre-conditional gene-trap mutation of *Smoc1* (*Smoc1^tm1a^*) that reduces mRNA to ∼10% of wild-type levels. This gene-trap results in highly penetrant hindlimb post-axial oligosyndactyly in homozygous mutant animals (*Smoc1^tm1a/tm1a^*). Eye malformations, most commonly coloboma, and cleft palate occur in a significant proportion of *Smoc1^tm1a/tm1a^* embryos and pups. Thus partial loss of Smoc-1 results in a convincing phenocopy of the human disease. SMOC-1 is one of the two mammalian paralogs of *Drosophila* Pentagone, an inhibitor of decapentaplegic. The orthologous gene in *Xenopus laevis*, *Smoc-1*, also functions as a Bone Morphogenic Protein (BMP) antagonist in early embryogenesis. Loss of BMP antagonism during mammalian development provides a plausible explanation for both the limb and eye phenotype in humans and mice.

## Author Summary

Ophthalmo-acromelic syndrome (OAS) is a rare congenital genetic disorder involving complete absence of the eyes and limb malformations, with missing or fused bones in the feet and hands. In this paper we report the identification of genetic changes to both copies of the *SMOC1* gene as the cause of most cases of OAS. We have identified eight different mutations in this gene in unrelated individuals, and six of these mutations are predicted to completely abolish SMOC-1 function. We have also genetically disrupted the mouse *Smoc1* gene to produce only 10% of normal levels. These animals, called *Smoc1^tm1a/tm1a^* mice, have similar hindlimb malformations to those seen in the limbs of human OAS patients, resulting in missing toes in some mice and fusion of toes in others. *Smoc1^tm1a/tm1a^* embryos and pups also have eye malformations but these are milder than those seen in human cases, perhaps because, unlike the human cases, the mice still have some residual function of the gene. We suggest that the normal function of SMOC-1 may be to regulate an important class of growth factors, called Bone Morphogenetic Proteins (BMPs), which are essential for normal embryonic development.

## Introduction

Congenital absence of an eye (here termed anophthalmia) is a rare malformation in humans with a live birth prevalence of less than 1 in 10,000 [1]. Identifiable single gene disorders account for ∼25% of bilateral anophthalmia. The known genetic causes include compound heterozygous mutations in *PAX6*, *de novo* heterozygous loss-of-function mutations in *SOX2* [2]–[4], inherited or *de novo* heterozygous loss-of-function mutations in *OTX2* [5], [6], homozygous loss-of-function mutations in *STRA6* [7] and possibly inherited, heterozygous loss-of-function mutations in *BMP4* [8]. In most cases of anophthalmia no eye is visible on clinical examination but optic nerves, chiasm and optic tracts remnants are visible on magnetic resonance imaging. Absence of the eye with ipsilateral absence of optic nerves, chiasm and optic tracts is termed true anophthalmia and is taken to suggest very early failure of ocular development.

**Figure 1:**
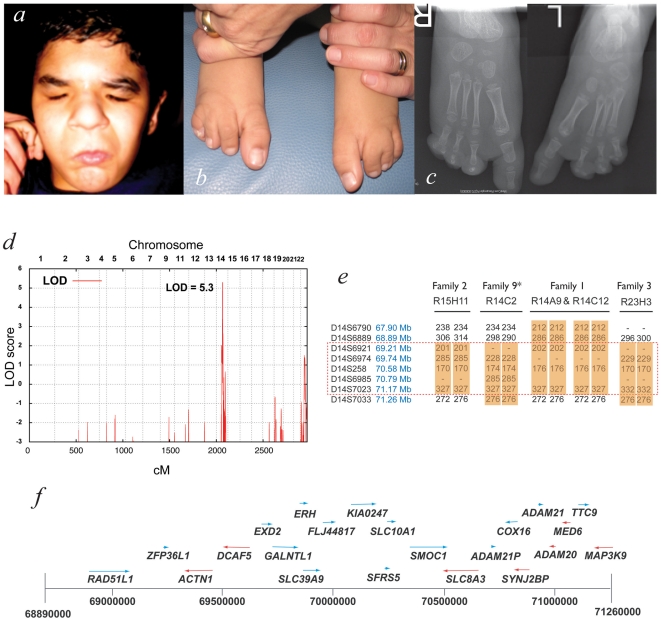
Mapping ophthalmo-acromelic syndrome. Clinical photographs (a,b) and radiographs (c) of patient R14C12 showing bilateral anophthalmia, in association with bilateral postaxial oligodactyly and cutaneous syndactyly of 2_nd_ & 3_rd_ toes. (d) Multipoint linkage analysis using 10K SNPchip data from families 1–3 showing a significant LOD score of Z = 5.3 at 14q22.3–24.2, a region also identified by autozygosity mapping (see Table 1). (e) Microsatellite marker analysis for affected individuals in Families 1–3 and Family 9 showing region of homozygosity, but no common haplotype. (f) The microsatellite data refined the OAS candidate interval to Chr14∶69.89–71.26 Mb which is shown diagrammatically with the 22 annotated genes that were sequenced in this study (UCSC assembly GRCh37).

Ophthalmo–acromelic syndrome (OAS), also known as Waardenburg anophthalmia syndrome, is one of the most frequently reported causes of true anophthalmia, which occurs in association with a distinctive pattern of distal limb anomalies (Figure 1a–1c). OAS is an autosomal recessive disorder (MIM #206920) first reported 50 years ago by Waardenburg in two unrelated families [9]. This original report illustrated the phenotypic spectrum associated with this disorder. The first family was a sibship of nine and consisted of two sisters with unilateral anophthalmia, one of whom had coloboma in her contralateral eye. Both had bilateral synostosis of the 4th and 5th metacarpals and bilateral postaxial four-toe oligodactyly with soft tissue syndactyly. A brother had similar limb involvement but with normal eyes and the other siblings were reported as normal. In the second family, the proband was a girl with significant learning disability, bilateral anophthalmia, short fingers bilaterally and postaxial oligodactyly with four toes on both feet. Her younger sister was normal. 32 further cases of OAS in 23 different families have been reported [10]–[29]. Of the reported definite cases; 32/35 (91.4%) had anophthalmia (4∶28 for unilateral:bilateral), 29 (82.9%) had lower limb postaxial oligodactyly, 20 (57.1%) had fusion of metacarpals 4–5 and 13 (37.1%) had learning disability. Other recurrent features included orofacial clefts (4/35) and perinatal or early postnatal death (10/35) in the 25 families. At the point of submission of this paper, very little was known of the molecular basis of OAS. A locus on 10p11.23 with a reported LOD score >3 had been suggested on the basis of linkage analysis in three unrelated families. However, no pathogenic mutations could be identified in any of the genes in the linkage interval [17].

Bone morphogenetic proteins (BMPs) account for 10 of the 33 members of the transforming growth factor beta (TGFβ) superfamily of peptide growth factors in humans and are encoded by the genes *BMP2-7*, *BMP8A*, *BMP8B*, *BMP10* and *BMP15* (*BMP1* does not encode a growth factor but a tolloid-like protease[30]). BMPs are secreted into the extracellular space where they bind to BMP type I serine-threonine kinase cell surface receptors encoded by *BMPR1A* and *BMPR1B*. The presence of BMP type I receptors appears sufficient for BMP binding, but a BMP type II receptor (encoded by *BMPR2*, *ACVR2A* and *ACVR2B*) is required for phosphorylation of the BMP type I receptors, endocytosis and activation of the signal transduction cascade [31]. The intracellular domain of the activated BMP type I receptors in turn phosphorylates a Ser-Ser-X-Ser (SSXS) motif at the C-terminal end of one of three homologous protein products of the human genes *SMAD1*, *SMAD5* and *SMAD9*. Phosphorylated SMAD1/5/9 (pSMAD1/5/9 known as regulatory- or R-SMADs) then bind to the co-SMAD encoded by *SMAD4.* The co-SMAD/R-SMAD complex then translocates to the nucleus where it functions as a transcription factor mediating the activation of target genes [32]. It has recently become clear that BMP signaling can also directly induce the activation of the MAPK pathway [33].

The formation of BMP signaling gradients is used extensively throughout vertebrate embryonic development. The formation and maintenance of stable developmental gradients appears to require multiple mechanisms to balance agonistic and antagonist effects on BMP signaling. The complexity of the system is demonstrated by the molecular basis of dorsal and ventral signaling centres in the gastrula of *Xenopus laevis* embryos [34]. The dorsal signaling centre (DSC; Spemann's organizer) has the general effect of antagonizing the Bmp gradient from the ventral signaling centre. The DSC secretes noggin and chordin, which (together with twisted-gastrulation [35]) bind to bmp in the extracellular space and prevents binding to the bmp type I receptor. The ventral signaling centre (VSC) secretes bmp4 and bmp7 but also bmper (bmp-binding endothelial regulator) [36] and sizzled, which inhibits tolloid-like 1, a zinc metalloproteinase that efficiently cleaves chordin [37]. The VSC also produces bambi (bmp and activin membrane-bound inhibitor), a bmp receptor that lacks the catalytic intracellular domain and thus acts dominant-negatively to inhibit bmp signaling [38].

SMOC-1 is encoded by the human gene *SMOC1* (*SPARC related modular calcium binding 1*) and was initially characterised as a basement membrane protein with significant homology to BM-40 (also known as SPARC and osteonectin) [39]. The domain structure of the SMOC-1 peptide and the close homolog SMOC-2 [40], is evolutionarily conserved [41] and consists from N- to C-terminus of a follistatin-like domain, two thyroglobulin type I (Tg1) domains and an EF-hand calcium-binding domain. The ortholog of SMOC-1 in *Xenopus laevis*, XSMOC-1, has been shown to function as a BMP antagonist. Uniquely among the known peptide BMP antagonists, SMOC-1 was able to antagonize BMP activity in the presence of a constitutively active BMP receptor. The molecular basis of this antagonism is not yet clear but may function by stimulating MAPK-mediated phosphorylation of the linker (i.e. non-SSXS) region of the R-SMAD proteins [42].

We report the identification of a locus for OAS on 14q24.2 with subsequent identification of homozygous, predicted loss-of-function mutations in the *SMOC1* gene in eight out of fourteen unrelated families with OAS. Whole mount *in situ* hybridisation (WISH) combined with optical projection tomography (OPT) shows site- and stage-specific developmental expression of the orthologous mouse gene, *Smoc1,* in embryonic limb bud and craniofacial structures. The phenotype associated with homozygosity for a targeted “pre-conditional” gene-trap mouse mutation of *Smoc1* also shows significant overlap with the human disease. SMOC-1 and SMOC-2 appear to be the two vertebrate paralogs of the *Drosophila* protein Pentagone that has recently been shown to function as an antagonist of Decapentaplegic (Dpp) signaling *in vivo* [33]. We discuss the potential role for SMOC-1 in modulating BMP signaling during eye and limb development.

## Results

### Mapping and Mutation Analysis

A locus for OAS at 14q24.2 was identified using autozygosity mapping with 10K SNP chip data from multiple, apparently unrelated consanguineous pedigrees. Affected individuals from eight of the fourteen families showed tracks of >20 homozygous SNPs in a row at this locus (Dataset S1). This locus was confirmed with multipoint linkage analysis using data from three of these families giving a combined LOD score of 5.3 (Figure 1d). The critical region was narrowed to ∼1 Mb using microsatellite markers in four families (Figure 1e). To identify the causative gene, all coding exons for each gene in the critical region were sequenced (Figure 1f).

**Figure 2:**
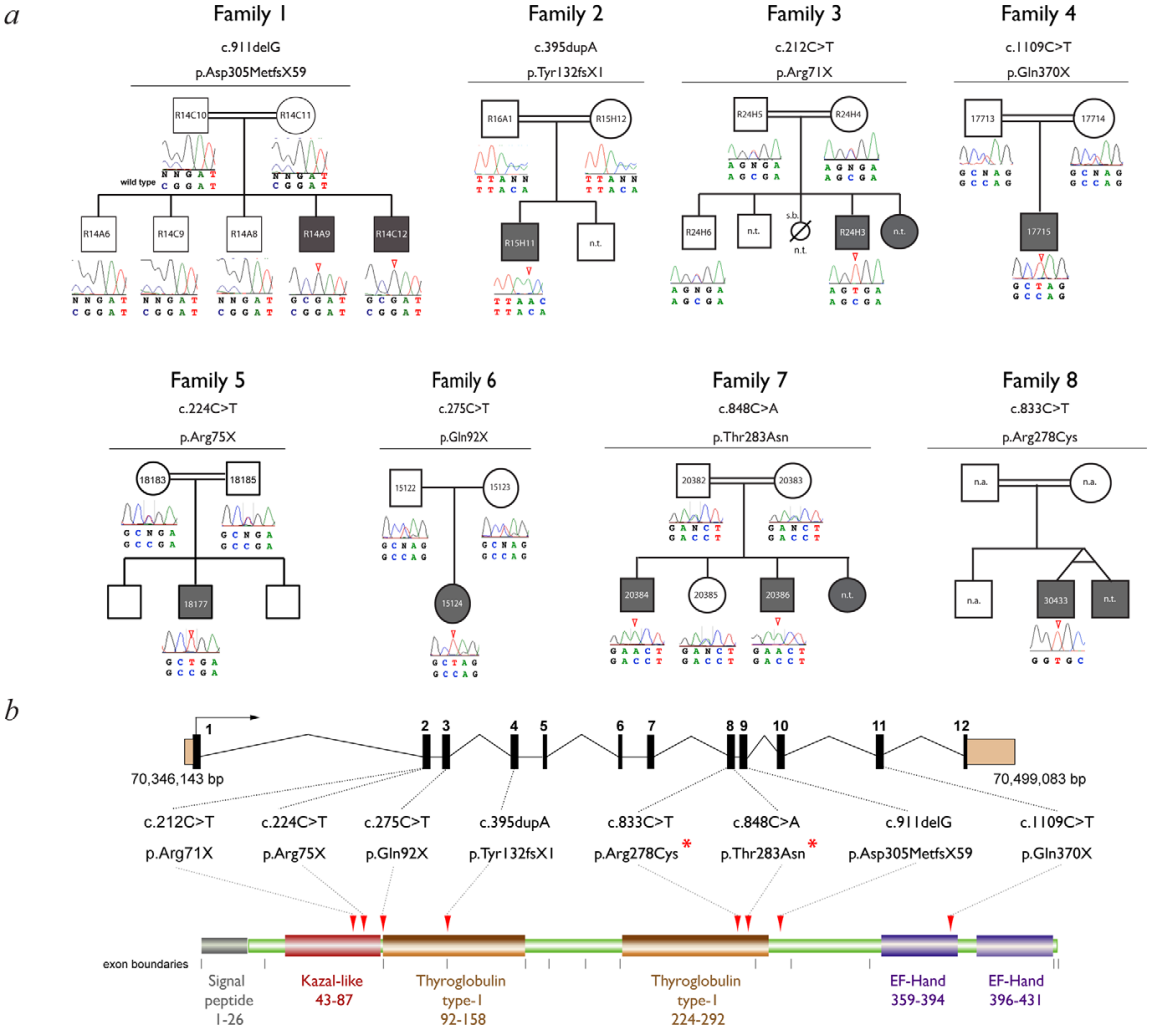
Mutation analysis. (a) Family pedigrees and associated *SMOC1* mutations identified. The pedigree for Family 1 is representative and shows segregation of a homozygous *SMOC1* mutation (c.911delG; p.Asp305MetfsX59) in affected individuals with both parents (and all unaffected sibs) being heterozygous carriers. (b) Schematic of the *SMOC1* gene (top) and predicted protein (below), illustrating the exon positions for all eight mutations identified in the OAS families. Coding exons are coloured black and numbered, UTRs are brown, protein domains are labeled with amino acid residue numbers. Red arrowheads indicate the position of the mutations in the peptide. Red asterisks highlight the missense changes, which are located in the second thyroglobulin domain thought to be involved in the control of proteolytic degradation (n.t.- Sample not tested).

Potentially deleterious mutations were identified in only one gene: *SMOC1,* and independent homozygous *SMOC1* mutations were found in eight out of fourteen families (Figure 2a, Table 1). Of these, 6 mutations predicted complete loss of protein function; 4 are nonsense mutations and 2 are single base deletions or insertions resulting in a frameshift. Two different missense changes were identified, both are in the C-terminal region of the second thyroglobulin type I domain of SMOC-1 (Figure 2b). No mutations were identified in sequence analysis of the *SMOC1* coding region in 190 healthy blood donors.

**Table 1:** Clinical features and mutations in affected individuals with Ophthalmo-Acromelic Syndrome.

FAMILY	1	2	3	4	5	6	7	8		
Affected Case	R14A9	R14C12	R15H11	R23H3	17715	18177	15124	20384	20386	30433
Published	Unpublished	Garavelli et al., (2006)	Khan & Zafar, (2008)	Suyugul et al, (1996) (case 3)	Pallotta & Dallapiccola, (1984)	Unpublished	Sayli et al. (1995)	Pallotta & Dallapiccola, (1984)		
Age assessed	13 Yr	9 Yr	6 Mo	7 Mo	18 Yr	40 Yr	11 Yr	14 Yr	7 Yr	10 Yr
Sex (Ratio)	M	M	M	F	M	M	F	M	M	M
Ethnicity	Lebanese	Lebanese	Gypsy	Pakistani	Turkish	Calabrian	Puerto Rican	Turkish	Sicilian	
Consanguinity	+	+	+	+	+	+	−	+	+	
Ocular defect	None	BA	BA	UA	BA	BA	BA	BA	BA	BA
Optic nerve/tract/chiasm present on scan?	−	Unknown	Remnants of optic nerve	Unknown	Absent	Absent	Unknown	Unknown	Unknown	Absent
Upper limb	cut synd	cut synd, hypopl 5^th^ finger	bilat 4/5 metacarpal fusion	−	bilat 4/5 metacarpal fusion, camptodactyly	bilat 4/5 metacarpal fusion	contractures of fingers	short 5^th^ metacarpals	short 5^th^ metacarpals	clinodactyly 5th fingers
Lower Limb	cut synd 3–5	bilat missing postaxial ray cut synd 2–4 right, 2/3 left	bilat missing postaxial ray	bilat missing postaxial ray	bilat missing postaxial ray	bilat missing postaxial ray	bilat missing postaxial ray & cut synd 2/3	Right fusion 4/5 metatarsal & phalanx, cut synd 2–5	cut synd toes 2–5	cut synd toes 4/5
Other Limb/Skeletal Defect			Bowed tibia		Contractures of elbows, Coxa valga		TEV, bowed tibias			
Craniofacial	−	Cleft palate	−	Pierre Robin Sequence						Highly arched palate
Other defects	Horseshoe kidney, hypospadias	Horseshoe kidney, mental retardation	Horseshoe kidney		Severe mental retardation, epilepsy, cryptorchidism	Severe mental retardation	Horseshoe kidney			Severe mental retardation
Coding change	c.911delG	c.911delG	c.395dupA	c.212C>T	c.1109C>T	c.224C>T	c.275C>T	c.848C>A	c.848C>A	c.833C>T
Protein change	p.Asp305MetfsX5	p.Asp305MetfsX5	p.Tyr132fsX1	p.Arg71X	p.Gln370X	p.Arg75X	p.Gln92X	p.Thr283Asn	p.Thr283Asn	p.Arg278Cys
Exon	9	9	4	2	11	2	3	8	8	8
Mutation Type	Frameshift 2	Frameshift 2	Frameshift 1	Nonsense 1	Nonsense 2	Nonsense 3	Nonsense 4	Missense 1	Missense 1	Missense 2
IBD 14q24.2	Yes	Yes	Yes	Yes	Yes	Yes	Yes	Yes	Yes	Yes

Yr = years; Mo = months; F = Female; M = Male; UA/BA = Unilateral/Bilateral anophthalmia; IBD = Identity by Descent; Cut synd = cutaneous syndactyly; TEV = talipes equinovarus; 2/3 = second and third digits; 3–5 = third, fourth and fifth digits; 2–4 = second third and fourth digits; 2–5 = second, third, fourth and fifth digits; 4/5 = fourth and fifth digits; bilat = bilateral.

No mutations in *SMOC1* could be identified in 6 of the 14 families. There were no obvious phenotypic differences between the cases with and without *SMOC1* mutations; all have classical OAS. SNP and microsatellite data on two of the six families without detectable mutations showed large regions of homozygosity across the 14q24 region containing *SMOC1.* This suggests that we have missed a mutation within the transcription unit or that there may be a regulatory mutation impairing *SMOC1* transcription. In the four remaining families no plausible locus could be identified using homozygosity mapping.

### Expression Analysis of *Smoc1*

**Figure 3:**
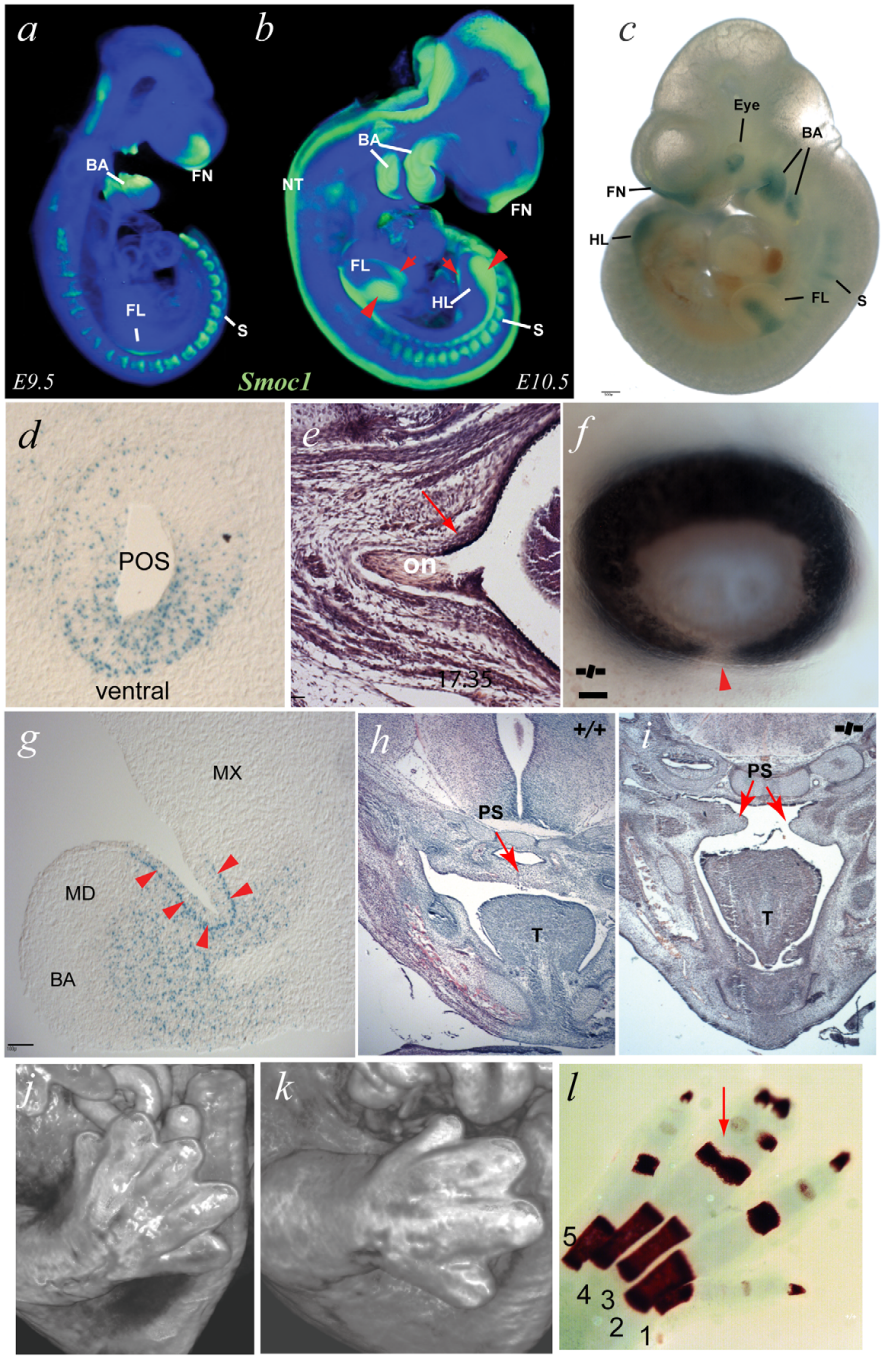
A targeted *Smoc1* mutation caused an ophthalmo-acromelic-like phenotype in mice. (a) OPT representation of wild type (WT) *Smoc1* expression at embryonic day (E) 9.5 (green represents *Smoc1* expression); *Smoc1* is expressed in the pharyngeal (branchial) arches (BA), the rostral neural tube (NT), in the anlage of the forelimbs (FL), the fronto-nasal region (FN), and in the somites (S). (b) At E10.5, expression is maintained in the branchial arches, somites and in the frontal nasal processes, as well as extending caudally in the neural tube. (c) In E10.5 *Smoc1^tm1a/tm1a^* embryos, β-galactosidase activity was observed in tissues consistent with the OPT analysis of WT *Smoc1* expression: in the dorsal hindlimbs; in the medial regions of dorsal and ventral forelimbs, the branchial arches, in the frontonasal processes, and in the somites. In addition, strong signal was observed in the eye region (scale bar = 500 µm). (d) X-gal stained sagittal sections of a representative E10.5 *Smoc1^tm1a/tm1a^* embryo in the developing eye showing that expression was restricted to ventral regions of the presumptive optic stalk (POS). (e) Examination of optic nerve morphology identified an extension of the RPE into the dorsal optic nerve in mutant animals compared to control. (f) Photographs of *Smoc1^tm1a/tm1a^* eye showing an optic fissure closure defect (arrowhead) consistent with coloboma (scale bars = 100 µm). (g) Expression in the 1^st^ branchial arch mesenchyme was distributed in proximal regions and absent from distal areas, with positive signal also seen in the epithelial cells at the hinge region between maxilliary (MX) and mandibular (MD) components (arrowheads) (scale bar = 100 µm). (h,i) Pictomicrographs of sections through E14.5 heads showing a failure in palatal shelf (PS) fusion in the developing palate in the *Smoc1^tm1a/tm1a^* embryo (i) compared to the fully fused WT littermate (h). (j,k) Surface rendered visualization of OPT reconstructions of hindlimbs at E14.5. (j) WT embryo with normal arrangement of 5 digits in the hindlimb whereas the *Smoc1^tm1a/tm1a^* littermate (k) had hindlimb oligodactyly affecting the axial digits, with only 4 digits present. (l) Skeletal preparation of P0 *Smoc1^tm1a/tm1a^* hindlimb with osseous fusion of the phalanges of digits 3–4 (red arrow).

As a first step to determining the likely developmental role of *SMOC1* we undertook developmental expression analysis of the orthologous gene *Smoc1* in whole mouse embryos. WISH analysis with an antisense riboprobe specific to *Smoc1* and optical projection tomography (OPT) were used to create a 3D representation of both the anatomy and colorimetric staining. We found site- and stage-specific expression of *Smoc1* at all stages examined (Figure 3a–3b; Video S1; Video S2). Staining in the limb bud was particularly interesting with expression seen first in the very early limb bud anlage from 9.5 dpc (Figure 3a). At 10.5 dpc the limb expression distinctly localised to both the dorsal and ventral surfaces of the forelimb, but was predominantly dorsal in the hindlimb bud (Figure 3b, Figure S1). Strong expression was seen in the developing pharyngeal arches and the frontonasal region with low-level expression in the ectoderm overlying the developing optic vesicle (Figure S1). Using WISH and OPT no clear expression of *Smoc1* was detected in the optic vesicle itself. There was clear expression in the developing somites at E9.5 and E10.5 (Figure 3a, 3b). At E9.5 there was also staining in the hindbrain (Figure 3a) and at E10.5 strong staining in the dorsal neural tube (Figure 3b).

### Targeted Mutation in Mouse *Smoc1*

In order to determine if the non-redundant developmental role of *SMOC1* is evolutionarily conserved we obtained mice with a targeted Pre-Conditional mutation in *Smoc1* containing a *LacZ* reporter allele created as part of the EUCOMM project [43]. The integrated location and the details of the targeting construct are shown in Figure S1d. The *Smoc1^tm1a/tm1a^* mice have ∼10% of wild-type levels of *Smoc1* mRNA during development (Figure S1f), presumably as a result of splicing across the gene-trap insertion.

Using the *LacZ* reporter in targeted animals, we were able to further assess the developmental expression of *Smoc1* (Figure 3c, 3d, and 3g). Spatial and temporal expression data were similar between *Smoc1^+/tm1a^* and *Smoc1^tm1a/tm1a^* genotypes, but with less intense staining in *Smoc1^+/tm1a^* mice (data not shown). The X-gal staining at E10.5 of both whole mount embryos and cryosections was consistent with the wild type OPT data. Cryosections through the maxilliary-mandibular hinge region of the 1^st^ pharyngeal (branchial) arch at E10.5 showed striking regional specificity of gene activation (Figure 3g). The expression was present within the mesenchyme or neural crest of both components of the first arch but is most intense in the sub-epithelial mesenchyme within the hinge region. Sharp boundaries of expression are evident in the subepithelial mesenchyme (Figure 3g). The only major difference between the riboprobe WISH analysis and the X-gal staining was the strong expression identified within the developing eye seen in the latter but not the former (Figure 3c). This staining was particularly strong at E10.5 within the ventral aspect of the developing optic nerve (Figure 3d).

Further phenotypic analysis of mutant animals showed that limb malformations were present in a high proportion of embryos and pups (Table 2). Oligodactyly (+/− fibular agenesis) or osseous syndactyly of both hindlimbs was present in 11/12 of *Smoc1^tm1a/tm1a^* animals (Figure 3j–3k and Figure S1). *Smoc1^tm1a/tm1a^* embryos and neonates were also smaller than littermates but did not appear to have any other major malformations (Figure S2). To date all *Smoc1^tm1a/tm1a^* animals allowed to litter have died at or soon after birth. A proportion of this perinatal lethality is likely be related to the presence of cleft palate in a significant proportion (4 of 12) of the *Smoc1^tm1a/tm1a^* animals (Figure 3i). However it is not clear what accounts for the mortality in the non-cleft cases. Interestingly, unexplained perinatal lethality is reported in human OAS families (see Introduction).

**Table 2:** Phenotypes identified in *Smoc1-*targeted mice.

Genotype	*Smoc1^tm1a/tm1a^*	*Smoc1^+/tm1a^*	*Smoc1^+/+^*
Total analysed	12 (26%)	21 (45.7%)	13 (28.3%)
Eye phenotype	5 (n = 9*; 55.6%)	0	0
Hind limb phenotype	11 (91.7%)	0	0
Cleft palate	4 (33.3%)	0	0
Missing fibula	6 (50%)	0	0

*3 confirmed *Smoc1^tm1a/tm1a^* animals were embedded in paraffin before eye phenotype was established.

Eye malformations were apparent in a significant proportion of the homozygous embryos or pups. We observed iris and retinal coloboma in 55.6% of the homozygous animals that could be accurately phenotyped (Figure 3f). Extension of the retinal pigmented epithelium (RPE) into the optic nerve (figure 3e), and a reduction in optic nerve diameters (Figure S2) was also observed in these mice.

## Discussion

We report compelling evidence that loss-of-function mutations in *SMOC1* cause a significant subset of OAS cases. No non-synonymous changes were found in any region of the *SMOC1* coding region in the 190 control individuals that were fully sequenced but we identified eight mutations within OAS families that are all different and homozygous. Six of these mutations (four nonsense and two frame-shift) are very likely to result in severe or complete abrogation of protein function. The two missense mutations (p.Arg278Cys & p.Thr283Asn) we have identified are both located in the second thyroglobulin type-1 domain (Tg1) of SMOC-1. Tg1 domains are cysteine-rich motifs that were first identified in the C-terminal region of thyroglobulin which appear to function as peptidase inhibitors, specifically inhibitors of cysteine cathepsins [44], [45]. Neither Arg278 or Thr283 show identity at the equivalent residue within the first Tg1 in human SMOC-1. However, both residues show complete conservation with the second Tg1 in both mouse and *Xenopus tropicalis* Smoc-1 and Thr283 is conserved in the second Tg1 in *Drosophila* Pentagone (see Figure S3). The mutation of Arg278 to Cys may disrupt the highly conserved pattern of disulphide bonding within the second Tg1 [41]. Given that there is no obvious difference between the missense mutation cases and those with null mutations, it is reasonable to speculate that inhibition of a developmentally expressed peptidase, possibly a cysteine cathepsin, may be the non-redundant developmental function of SMOC-1. Interestingly, Cathepsin H has been shown to be involved in Bmp4 degradation during lung development [46]. It is also possible that a mutation resulting in constitutive activation of the target peptidase could phenocopy *SMOC1* mutations.

The expression analysis and targeted partial gene inactivation in mouse embryos strongly supports the critical and non-redundant developmental role of SMOC-1 suggested by the human genetic analysis and that this role is conserved across evolutionary time. The hindlimb phenotype in *Smoc1^tm1a/tm1a^* homozygous mice was very similar to the lower limb phenotype in human OAS cases. The combination of osseous syndactyly and oligodactyly suggest that the mechanisms controlling digit number within the limb bud are significantly impaired. The control of digit number is critically dependent on correct dosage of sonic hedghog (Shh) [47], [48] and BMP4 & BMP7 signaling proteins [49], [50]. A significant proportion of the *Smoc1^tm1a/tm1a^* homozygous mice have cleft palate, a feature common to human OAS and consistent with the high level of expression of *Smoc1* that was detected in the developing first pharyngeal arch. The eye malformations seen in the mouse were less severe than those seen in human OAS cases, being predominantly iris and retinal coloboma. This may relate to the difference in mutation type between the mouse and human cases: most human mutations are apparently null, whereas the mouse line had 10% of normal *Smoc1* transcript levels present, most likely due to splicing across the gene trap. This level of Smoc-1 function could partially rescue the ocular phenotype in the mice. Analysis of the phenotype associated with a null allele in mice that we are currently making will answer this question. We will also screen a cohort of human patients to determine if hypomorphic mutations in *SMOC1* may cause coloboma.

The formation of precise gradients of diffusible ligands is required during embryogenesis both for patterning - the formation of complex tissue structures from apparently homogenous populations of multipotent cells - and to control growth to achieve correct final organ size with appropriate symmetry of paired structures. BMPs represent an important class of diffusible ligands with roles in both patterning and control of organ size [51], [52]. Much of what we know about the formation, maintenance and function of BMP gradients derived from studies of *Drosophila* Decapentaplegic (Dpp) [53]. BMPs are considered to be the mammalian paralogs of Dpp. SMOC-1 and its close homolog SMOC-2 appear to be the mammalian paralogs of *Drosophila* Pentagone (Pent) [54]. Pent functions as an *in vivo* antagonist of Dpp by preventing receptor endocytosis close to its source thus allowing gradients to form over wider distances within the wing imaginal discs. Lack of Pent results in a very steep and narrow gradient of Dpp signaling, which in turn causes a relative deficiency of Dpp further from the source. In *Drosophila* the control of cell proliferation within the wing imaginal disc is dependent on Dpp signaling [55]. In the chick it has been shown that the level of cell proliferation within the limb bud must be precisely specified to in order to result in sufficient antero-posterior expansion to form the correct digit number [48]. If a similar mechanism exists in vertebrates then it may be that *SMOC1/Smoc1* mutations could cause oligodactyly by altering the BMP gradient within the limb bud and thus alter anteroposterior expansion. Although the molecular basis of the developmental pathology associated with OAS remains to be elucidated, support for SMOC-1 mediated BMP antagonism as a component is provided by human and mouse genetic data that indicate the importance of BMP signalling in both limb [56], [57] and eye [8], [58], [59] development. Interestingly, heterozygous *BMP4* mutations have been associated with microphthalmia, microcornea, coloboma, retinal dystrophy, and tilted optic disc [8]. In addition, *BMP4* mutations are also associated with digital anomalies (polydactyly) and cleft lip/palate [60]. The partial overlap between the OAS phenotype and the phenotypes associated with BMP4 disruptions may reflect a functional relationship between SMOC-1 and BMP4.

Following submission of this paper two other groups have identified *SMOC1* mutations as a cause of OAS [10], [61]. One group studied five affected individuals from four unrelated OAS families [61]. They identified the locus on 14q24 and then found *SMOC1* mutations in three out of four of the families. Interestingly these were the same families in whom linkage to 10p11.23 had been previously reported by the same group [17]. The *SMOC1* mutations were all homozygous and plausibly loss of function (one nonsense and two 5′ splice site mutations). This group also reported the phenotype in homozygous mice with Sleeping beauty transposon-induced gene trap mutations of *Smoc1*. The expression analysis and limb phenotype of the mice are very similar to that reported here. Their homozygous mice also showed unexplained uniform early lethality. Interestingly their mice had small eyes but they do not report coloboma. The optic nerves were shown to be significantly narrower than non-homozygous animals and they also showed extension of the RPE into the optic nerve. The second paper reports linkage to 14q24 and identification of a 5′ splice site mutation in *SMOC1* in a single multiplex family with OAS [10]. This group also reports developmental expression analysis of the orthologous gene, *smoc1*, in zebrafish embryos. Expression was evident in the brain, choroid fissure and pharyngeal arches. “Knock-down” experiments using a morpholino targeted to *smoc1* resulted in microphthalmia and brain abnormalities in the injected embryos. Taken together these papers strongly support loss of SMOC-1 function as the major cause of OAS and that this protein has a conserved non-redundant function during ocular and limb development.

Finally, in six families with typical OAS we could not identify *SMOC1* mutations, including the original family described by Waardenburg in 1935 [9]. In two of these six families, affected individuals show homozygosity over the region of 14q24.2 suggesting that we have significant limitations in our current *SMOC1* mutation analysis strategy. However, four families showed no apparent autozygosity around *SMOC1,* suggesting the likely existence of other OAS loci. Identifying causative genes at other loci is likely to help elucidate the embryopathology and is an active area of our future work.

## Materials and Methods

### Patient Recruitment and Ethics Approval

All patient related work was carried out with full written consent of the families. Details of the mutation positive cases are provided in Table 1. The informed consent process was reviewed and approved following consideration by national ethical committee systems in the UK and the Netherlands. Several of the cases have been previously published. [16], [19], [22], [25], [26]

### Mapping and Linkage Analysis

Patient, parental and unaffected sib genomic DNA samples were run on Affymetrix GeneChip Human Mapping 10K Arrays (Xba131) and autozygosity mapping was performed using ExludeAR 1 with data subjected to multipoint linkage analysis using ALOHOMORA and Allegro (V1.2c). Gene data in the candidate interval were retrieved from the Ensembl Human genome (GRCh37 assembly; http://www.ensembl.org/Homo_sapiens/index.html). Microsatellites containing tri- or tetranucleotide repeats (Table 3) were identified from the UCSC browser (http://genome.ucsc.edu/index.html) and PCR primers were designed using Primer3 (http://frodo.wi.mit.edu/primer3/). FAM fluorescent labels were placed at the 5′-end of the forward primer. All microsatellites were tested for informativeness for each family.

**Table 3:** Microsatellite repeat marker PCR and primer properties.

Microsatellite (Chr14: bp)	Forward primer	Reverse primer	Approx size (Repeat type)
D14S6790 (67906158–67906384)	CTGACAATTTGGGGAAAAGG	GGTACTGCTCTGAGGTCTGGA	320 bp (tetra)
D14S6889 (68892553–68892831)	TCTGGAGGACTCAGAAGAAGAGA	CCCAGGCAACAAGAGTGAA	300 bp (tetra)
D14S6921 (69216842–69217056)	CAGCTACTTCCACCGTCTCC	ACACTTGGTGCCCTTGAAAC	200 bp (tetra)
D14S6974 (69749225–69749449)	CGCCCTTGGAAATGATTTTT	GATAGCACCACTGCACTCCA	250 bp (tetra)
D14S258 (70583011–70583186)	TCACTGCATCTGGAAGCAC	CTAACTAAATGGCGAGCATTGAG	170 bp (di)
D14S6985 (70786661–70786943)	CTCCATGAAACACCCAGTCC	GGCAGAAAAATCGCTTGAAC	285 bp (tetra)
D14S7023 (71168266–71168625)	AGGGGTTAGCGAGAAGGAAG	GCAGGTAGAGGATGCCAGAG	330 bp (tri)
D14S7033 (71259290–71260458)	TGAGCCCAGGAGTTCAAGAC	AGCTCCCAGCATAGTTCCAG	270 bp (tetra)

### PCR and Sequence Analysis

Genomic DNA samples were Whole Genome Amplified (WGA) using the Illustra GenomiPhi V2 DNA Amplification Kit (GE Healthcare) according to the manufacturer's guidelines. For PCR, 50 ng WGA template DNA was amplified with 0.2 µM primers and 2x Custom Reddymix (Thermo Scientific) in H_2_O to 25 µl. Cycle conditions: 95°C×3 minutes; and 35×cycles of 95°C×1 minute, 56°C×45 seconds, and 72°C×1 minute; followed by a single step of 72°C×10 minutes. All mutations were confirmed by resequencing using non-WGA genomic DNA with identical reaction conditions. Sequencing was performed using Applied Biosystems 3130/3170 Genetic Analysers. Genbank sequences were downloaded from NCBI build 37.2 and mutation analysis was performed with Mutation Surveyor Software (SoftGenetics LLC, PA, USA) or Sequencher 4.8 (GeneCodes Corp. MI, USA).

### In Situ Hybridisations

To generate a DNA tempate for production of *Smoc1* riboprobe, PCR was performed from mouse genomic DNA targeting the 3′ untranslated region (UTR) of the *Smoc1* gene using primers with T3 and T7 RNA polymerase sites at the 5′ ends of the forward and reverse primers respectively (underlined) (*Smoc1* Forward 5′- AATTAACCCTCACTAAAGGCGTGTGTGGTTTGTTTCTGG-3′; *Smoc1* Reverse 5′- TAATACGACTCACTATAGGTAGACTGCCAAGGGATCTGG-3′). Digoxigenin (DIG) labelled (Roche) sense and antisense riboprobes were generated by *in vitro* transcription using T3 and T7 RNA polymerase respectively. Whole-mount in situ hybridization to mouse embryos at 9.5 & 10.5 days post-coitum (dpc or E9.5/E10.5) was carried out as previously described [62]. Briefly embryos were fixed overnight in 4% paraformaldehyde (PFA) at 4°C, proteinase K (10 µg/ml) (Roche) treated for 15–35 minutes, depending on the stage then washed twice in 0.1 M triethanolamine, washed in phosphate-buffered saline (PBS) -Tween (0.1%) and refixed in 4% PFA/0.2% glutaraldehyde for 20 minutes. Prehybridisation was performed for 2 hours and hybridisation for 40 hours at 60°C in hybridisation buffer containing each DIG labelled probe. Washes were in 2×hybridisation buffer for 10 minutes, 3×2×SSC +0.1% Tween 20 for 20 minutes, 3×0.2×SSC +0.1% Tween 20 for 30 minutes, all at 60°C. At room temperature the embryos were washed in 1 M Malic Acid with 2% BMB (Boehringer- Mannheim blocking reagent) +20% heat-treated lamb serum solution for 2 hours and then in the same buffer containing a 1/2000 dilution of anti-DIG antibody coupled to alkaline phosphatase (Roche) overnight at 4°C. Embryos were washed 3×5 minutes and 3×1 hour in MAB. Colour detection was performed in 2 ml of BM purple precipitating solution (Roche).

### *Smoc1* Targeted Mouse

The *Smoc1* pre-conditional knockout mouse (EUCOMM Project 48154; strain C57BL/6N-A-Smoc1^tm1a(EUCOMM)WTSI^, referred to as *Smoc1^tm1a^* in this manuscript) was generated by the International knockout Mouse Consortium (IKMC) under UK Home Office Project License 60/3785 (IJ Jackson, MRC Human Genetics Unit). Details of the allele and targeting strategy can be found at: http://www.eucomm.org/htgt/report/gene_report?project_id=48154 and in Figure S1. Genotypes were confirmed using the *Smoc1* forward primer 5′-GGTCTGACTCGGTAGGCTTG- 3′ (positioned at mChr12∶82,236,250 bp) and *Smoc1* reverse primer 5′-CCTCTCTCCAACCCTTTTCC-3′ (positioned at mChr12∶82,236,907 bp) which flank the targeted endogenous exon and produce a wild type PCR amplicon of 658 bp. Adding the targeting cassette specific primer 5′- TTAGTCCCAACCCCTTCCTC-3′ to PCR mixes as multiplex reactions produced a targeted-allele specific amplicon of 250 bp. Ear-clip DNA was extracted by incubating in 50 µl of 25 mM NaOH 0.2 mM EDTA solution at 95oC for 1 hour, then adding 50 µl of 40 mM Trizma. 1 µl of template was used for PCR reactions with 0.2 µM primers and 2x Custom Reddymix (Thermo Scientific) in H_2_O to 50 µl. Cycle conditions: 95°C×3 minutes; and 31×cycles of 95°C×45 seconds, 56°C×40 seconds, and 72°C×1 minute; followed by a single step of 72°C×10 minutes. Products were run on 1% TBE agarose gels. For qRT-PCR, mouse hind limbs at stage 10.5 dpc were dissected and mRNA extracted using TRIzol (Invitrogen) according to the manufacturers instructions. Mice were genotyped by PCR using genomic DNA as described above with WT and *Smoc1^tm1a/tm1a^* samples carried on for testing. Samples were DNaseI treated and then cycled using Power SYBR Green RNA-to-CT 1-Step Kit (Applied Biosystems) using an ABI-HT7900 SDS instrument (Applied Biosystems) with the following conditions: 48°C×30 minutes, 95°C×10 minutes, followed by 40 cycles of 95°C×15 seconds and 60°C×1 minute. Each 10 µl reaction contained 0.08 µl of RT Enzyme Mix; 5 µl of RT-PCR Mix; 1 µl RNA sample and 1.92 µl sdH_2_O, with the inclusion of either *Smoc1* forward 5′-GGATGGTTCCTTCACACAGG-3′ and reverse 5′- TCATCTCCATCGAACACAGG-3′ primers or *Hprt* forward 5′- CTGGTGAAAAGGACCTCTCG -3′ and reverse 5′- CAAGGGCATATCCAACAACA-3′ primers. Each reaction was performed in triplicate in optical reaction plates (384-well, Applied Biosystems), and RNA samples were also run without RT Enzyme Mix for negative controls with the same reaction conditions.

### Histological and Histochemical Analysis

Embryos were fixed in 4% PFA; dehydrated through graded alcohol series and xylene; and embedded in paraffin. Microtome sections were cut at 6 µm and rehydrated through ethanol series and stained with haematoxylin and eosin. For skeletal preparations the animals were dehydrated in 95% ethanol for 24 hours; followed by 72 hours in 100% acetone; 3 days in stain solution (1 part 0.3% alcian blue in 70% ethanol; 1 part 0.1% alazarin red in 95% ethanol; and 1 part acetic acid, in 17 parts 70% ethanol); followed by 3 days clearing in 1% KOH; 3 days in 1% KOH/30% glycerol; and two 24 hour periods in 1% KOH/50% glycerol; in 1% KOH/70% glycerol and were analysed in 100% glycerol. X-gal (5-bromo-4-chloro-3-indolyl-β-D- galactopyranoside) staining was performed as follows: targeted mouse embryos were dissected at 10.5 dpc and rinsed in PBS, then fixed for 1 hour in 4% PFA at 4oC, rinsed again in PBS and then washed for 3x 20 minutes in detergent wash (2 mM MgCl_2_, 0.1% Sodium Deoxycholate, 0.02% NP-40 [Igepal CA 630], in PBS). Detection was performed in β-galactosidase stain (0.085% NaCl, 5 mM K3 [Fe(CN)6], 5 mM K4 [Fe(CN)6], 200 µl/ml X-gal (Promega), in detergent wash), followed by a brief final stain fixation in 4% PFA for 30 minutes. For cryosection analysis, embryos were dissected and fixed as above, then incubated overnight in 20% sucrose/PBS at 4°C, transferred into OCT solution and frozen embedded on dry ice. Sections of 25 µm thickness were cut at −20°C, air dried and rinsed briefly in PBS. X-gal staining was then performed as described above.

### Optical Projection Tomography

For optical projection tomography (OPT) analysis *In Situ* stained embryos were mounted in 1% agarose, dehydrated in methanol and then cleared overnight in BABB solution (1 part Benzyl Alcohol: 2 parts Benzyl Benzoate). Samples were then imaged using a Bioptonics OPT Scanner 3001 (Bioptonics, UK) using brightfield analysis to detect tissue autofluorescence for capture of anatomical and signal data (wavelengths: excitation at 425 nm, emission: 475 nm). The resulting data were reconstructed using Bioptonics proprietary software (Bioptonics, MRC Technology, Edinburgh, UK), then automatically thresholded to remove background and finally merged into a single 3D image output using Bioptonics Viewer software.

## Supporting Information

Figure S1 Expression of Smoc1 in the limb buds and eye, and phenotype in Smoc1^tm1a/tm1a^ limbs. (a–c) Magnified images and digital section of the same OPT reconstruction of a wild-type E10.5 embryo shown in Figure 2 with green staining representing *Smoc1* expression. (a) *Smoc1* expression is seen in both the dorsal and ventral surface of the forelimb buds. (b) In the hindlimb expression is predominantly dorsal and with small region of ventral expression. (c) Digital section showing symmetrical low-level expression of *Smoc1* in the surface ectoderm overlying the optic vesicles (OV). (d) Diagram illustrating the gene targeting for the EUCOMM pre-conditional *Smoc1* knockout mouse at the genomic level (top) with the relative positions of RT-PCR primers indicated. Targeting cassette (bottom) is specific for exon 4 of *Smoc1*. Positions of genotyping PCR primers are indicated (GT-F & -R2, blue, are endogenous-gene specific, GT-R1 is cassette-specific) Note that the targeting cassette has the β-galactosidase ORF inserted downstream of an EN2 splice acceptor site (EN2 SA) and internal ribosomal entry site (IRES), allowing for reporter expression in successfully *Smoc1*-targeted animals (pA is Poly(A) tail). The cassette also features FRT and Cre-recombinase (loxP) sequences for future gene manipulation. Ensembl exon identifiers are given. A table of the phenotypes associated with the targeted allele is available in Table 2 of the main manuscript. (e) Agarose gel indicating each allele identified by genotyping PCR primers. A = phenotypically wild type animal, B = littermate with hindlimb oligodactyly, Wt = wild type unrelated mouse DNA sample. Primer pair 1 (GT-F & GT-R2) amplified wild type allele only, Primer pair 2 (GT-F & GT-R1) amplified the targeted allele only, multiplex reaction (GT-F & GT-R2 & GT-R1) identified both WT and mutant alleles and revealed animal B as a *Smoc1* homozygote (*Smoc1^tm1a/tm1a^*) and A as a heterozygote (*Smoc1^+/tm1a^*). Multiplex reactions were then used as standard for genotyping *Smoc1* status in analysed animals. (f) Quantitative RT-PCR analysis of dissected hindlimb tissue from E10.5 wild type and homozygote animals revealed significant reduction of Wt *Smoc1* mRNA in mutant animals (WT, n = 2; *Smoc1^tm1a/tm1a^*, n = 4). (g) Skeletal preparation of P0 *Smoc1^tm1a/tm1a^* hindlimb showing absent fibula (arrow) (T- tibia; F-femur).
(TIF)

Figure S2 Phenotypic analysis and X-gal staining of *Smoc1* mutant animals. (a & b) Low power magnification of H&E stained eyes revealed that mutant eyes were grossly normal but that overall eye size was reduced. (c & d) Higher magnification analysis revealed normal organization and retinal cell-layer lamination in *Smoc1^tm1a/tm1a^* mutant eyes. Abbreviations: c, cornea; ln, lens; r, retina; gcl, ganglion cell layer; inl, inner nuclear layer; onl, outer nuclear layer; rpe, retinal pigmented epithelium (Scale bars: a & b = 250 µm; c, d & e = 100 µm). (e) Maximum width measurements were taken for optic nerves (on) of *Smoc1^tm1a/tm1a^* mutants (*n = 17*) and controls (*n = 11*) from paraffin-embedded sections (f) This plot shows the significantly smaller mean diameter of the optic nerve in mutants compared to control animals with the error bars representing the non-overlapping of 95% confidence limits; 125.7 µm (118.2–133.3) and 147.0 µm (139.6–154.5) respectively. (g,h) Transverse digital sections from OPT scans of E14.5 WT and *Smoc1^tm1a/tm1a^* embryos showing an apparently normal size and shape of developing kidneys (arrows). Scale bar = 1 mm. (i) Whole mount X-gal stained *Smoc1^tm1a/tm1a^* embryo at E14.5. This embryo was dissected to reveal the developing kidneys. No X-gal positive cells can be seen in the kidneys (LK, left kidney; RK, right kidney) or bladder (B). Stain is clearly seen in both adrenal glands (arrowheads) and in tissue adjacent to the developing vertebrae (V). Scale bar = 500 µm. (j) Sagittal cryosection of 25 µm thickness counterstained with eosin showing strong X-gal staining in the tissue between the developing vertebral bodies (VB). (k) E14.5 stage left kidney seen in i. No stain positive cells can be seen within the kidney (KID) but a cluster of positively staining cells are seen within the adrenal (ADR). Scale bar = 100 µm.
(TIF)

Figure S3 Alignment of human SMOC-1 Thyroglobulin type-1 (Tg1) domains with Tg1 domains from mouse Smoc-1, Xenopus tropicalis Smoc-1 and Drosophila melanogaster Pentagone. Alignment of the Tg1-1 and Tg1-2 domains from mouse Smoc-1 and human SMOC-1 with the Tg1-2 domains from *Xenopus tropicalis* Smoc-1 and *Drosophila melanogaster* Pentagone. The position of identical amino acid residues across all sequences is given by pink shading. The gray shading indicates the conservation of the positions in the Tg1-2 affected by the missense mutations and the nature and position of the mutations is shown in red text below. Key: Tg1 = Thyroglobulin type-1 domain; Tg1-1 = first Tg1 in the peptide; Tg1-2 = second Tg1 in the peptide; hSMOC1 = human SMOC1; mSmoc1 = mouse Smoc1; xtSmoc1 = *Xenopus tropicalis* Smoc1; dmPent = *Drospophila melanogaster* Pentagone protein; Q9h4F8 etc are UniProt accession numbers.
(DOC)

Video S1 OPT analysis of *Smoc1* expression in wild-type E9.5 mouse embryo.
(MPG)

Video S2 OPT analysis of *Smoc1* expression in wild-type E10.5 mouse embryo.
(MPG)

Dataset S1 UCSC custom track for the hg18 genome build that represents the distribution of homozygous regions in the individuals affected with Ophthalmo-acromelic syndrome in our study. The homozygous regions are defined as the genomic coordinates encompassing 20 contiguous homozygous genotypic calls. The regions surrounding SMOC1 shows the multiple overlapping homozygous regions in unrelated families and provides a graphical representation of the linkage of OAS to this region of chromosome 14.
(TXT)
