# Correction: Loss of the BMP Antagonist, SMOC-1, Causes Ophthalmo-Acromelic (Waardenburg Anophthalmia) Syndrome in Humans and Mice

**DOI:** 10.1371/journal.pgen.1007866

**Published:** 2018-12-26

**Authors:** Joe Rainger, Ellen van Beusekom, Jacqueline K. Ramsay, Lisa McKie, Lihadh Al-Gazali, Rosanna Pallotta, Anita Saponari, Peter Branney, Malcolm Fisher, Harris Morrison, Louise Bicknell, Philippe Gautier, Paul Perry, Kishan Sokhi, David Sexton, Tanya M. Bardakjian, Adele S. Schneider, Nursel Elcioglu, Ferda Ozkinay, Rainer Koenig, Andre Mégarbané, C. Nur Semerci, Ayesha Khan, Saemah Zafar, Raoul Hennekam, Sérgio B. Sousa, Lina Ramos, Livia Garavelli, Andrea Superti Furga, Anita Wischmeijer, Ian J. Jackson, Gabriele Gillessen-Kaesbach, Han G. Brunner, Dagmar Wieczorek, Hans van Bokhoven, David R. FitzPatrick

It has come to the authors’ attention that there is a systematic numbering error in the cDNA nomenclature used to describe the causative genotypes. The authors are issuing a correction to rectify this mistake. This error does not affect the scientific accuracy of the underlying data.

Fig 2 and its legend have been corrected to account for this change. The corrected cDNA and protein numbers in Fig 2 are shown in red text.

**Fig 2:**
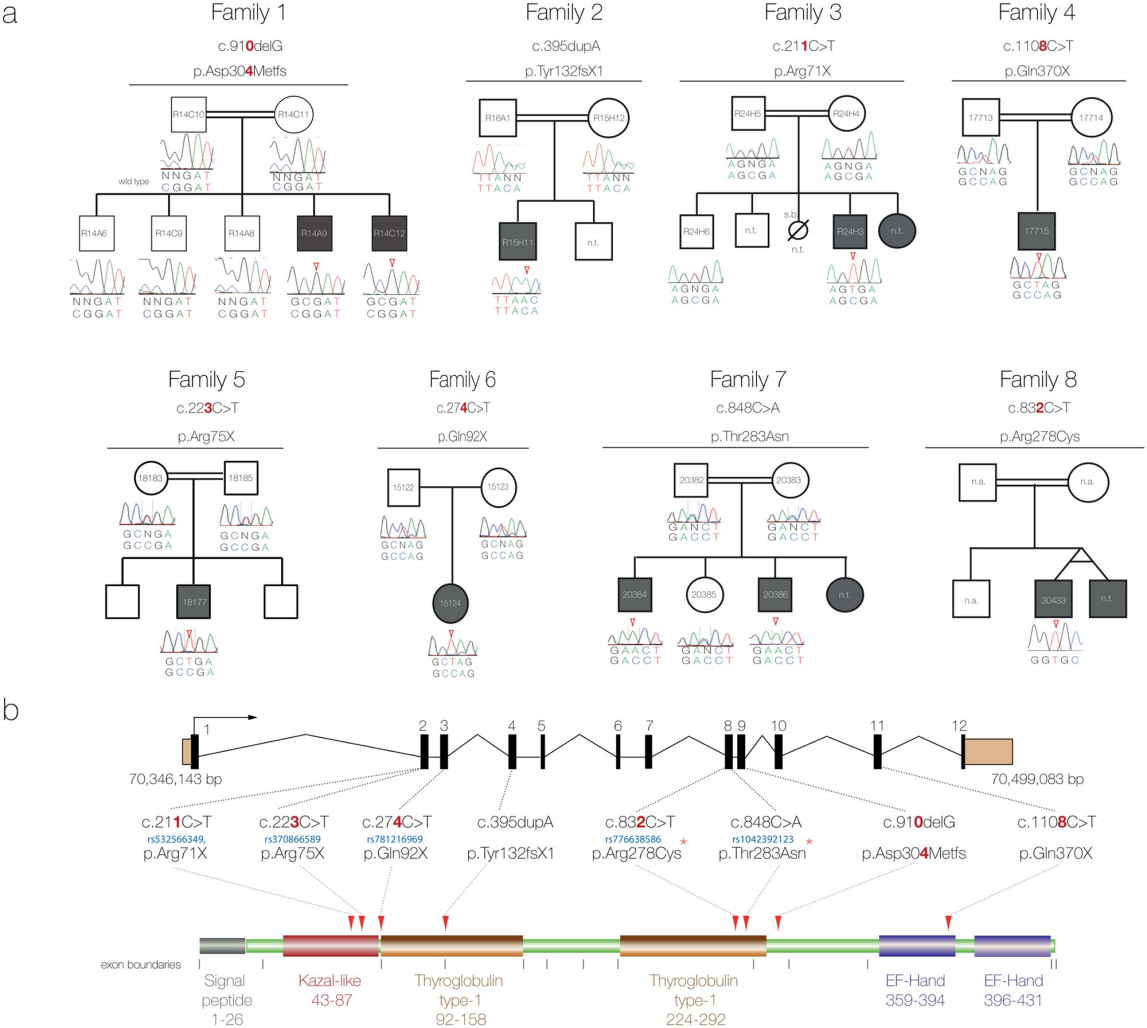
Mutation analysis. (a) Family pedigrees and associated *SMOC1* mutations identified. The pedigree for Family 1 is representative and shows segregation of a homozygous *SMOC1* mutation (c.910delG; p.Asp304Metfs*60) in affected individuals with both parents and the unaffected siblings being heterozygous carriers. (n.t.- Sample not tested). (b) Schematic of the *SMOC1* gene (top) and predicted protein (below), illustrating the exon positions for all eight mutations identified in the OAS families. Coding exons are coloured black and numbered, UTRs are brown, protein domains are labeled with amino acid residue numbers. Red arrowheads indicate the position of the mutations in the peptide. Where available the dbSNP rs numbers of the variants are provided in blue text. Red asterisks highlight the missense changes, which are located in the second thyroglobulin domain thought to be involved in the control of proteolytic degradation. The Ensembl transcript ENST00000361956 was used to position the variants in the cDNA.

Table 1 has also been corrected. The corrected cDNA and protein numbers are shown in bold red text.

**Table 1:** Clinical features and mutations in affected individuals with Ophthalmo-Acromelic Syndrome.

FAMILY	1	2	3	4	5	6	7	8		
Affected Case	R14A9	R14C12	R15H11	R2**3**H3	17715	18177	15124	20384	**20386**	**30433**
Published	Unpublished	Garavelli et al., (2006)	Khan & Zafar, (2008)	Suyugul et al, (1996) (case 3)	Pallotta & Dallapiccola, (1984)	Unpublished	Sayli et al. (1995)	Pallotta & Dallapiccola, (1984)		
Age assessed	13 Yr	9 Yr	6 Mo	7 Mo	18 Yr	40 Yr	11 Yr	14 Yr	7 Yr	10 Yr
Sex (Ratio)	M	M	M	F	M	M	F	M	M	M
Ethnicity	Lebanese	Lebanese	Gypsy	Pakistani	Turkish	Calabrian	Puerto Rican	Turkish	Sicilian	
Consanguinity	+	**+**	**+**	**+**	**+**	**+**	**-**	**+**	**+**	
Ocular defect	None	BA	BA	UA	BA	BA	BA	BA	BA	BA
Upper Limb	cut synod	cut synd, hypopl 5^th^ finger	bilat 4/5 metacarpal fusion	**-**	bilat 4/5 metacarpal fusion, camptodactyly	bilat 4/5 metacarpal fusion	contractures of fingers	short 5^th^ metacarpals	short 5^th^ metacarpals	clinodactyly 5th fingers
Lower limb	Cut synod 3–5	Bilat missing postaxial ray cut synd 2–4 right, 2/3 left	Bilat missing postaxial ray	Bilat missing postaxial ray	Bilat missing postaxial ray	Bilat missing postaxial ray	Bilat missing postaxial ray & cut synd 2/3	Right fusion 4/5 metatarsal & phalanx, cut synd 2–5	cut synd toes 2–5	cut synd toes 4/5
Other Limb / Skeletal Defect			Bowed tibia		Contractures of elbows, Coxa valga		TEV, bowed tibias			
Craniofacial	**-**	Cleft palate	**-**	Pierre Robin Sequence						Highly arched palate
Other Defects	Horseshoe kidney, hypospadias	Horseshoe kidney, mental retardation	Horseshoe kidney		Severe mental retardation, epilepsy, cryptorchidism	Severe mental retardation	Horseshoe kidney			Severe mental retardation
Coding change (homozygous)	c.910delG	c.910delG	c.395dupA	c.211C>T	c.1108C>T	c.223C>T	c.274C>T	c.848C>A	c.848C>A	c.832C>T
Protein change	p.Asp304Metfs*60	p.Asp304Metfs*60	p.Tyr132**X**	p.Arg71X	p.Gln370X	p.Arg75X	p.Gln92X	p.Thr283Asn	p.Thr283Asn	p.Arg278Cys
Exon	9	9	4	2	11	2	3	8	8	8
Mutation Type	Frameshift	Frameshift	Frameshift	Nonsense	Nonsense	Nonsense	Nonsense	Missense	Missense	Missense
IBD 14q24.2	Yes	Yes	Yes	Yes	Yes	Yes	Yes	Yes	Yes	Yes

Yr = years; Mo = months; F = Female; M = Male; UA/BA = Unilateral/Bilateral anophthalmia; IBD = Identity by Descent; Cut synd = cutaneous syndactyly; TEV = talipes equinovarus; 2/3 = second and third digits; 3–5 = third, fourth and fifth digits; 2–4 = second third and fourth digits; 2–5 = second, third, fourth and fifth digits; 4/5 = fourth and fifth digits; bilat = bilateral.
