# Supplementary figures and images for: Loss of the BMP Antagonist, SMOC-1, Causes Ophthalmo-Acromelic (Waardenburg Anophthalmia) Syndrome in Humans and Mice
Source: PLoS Genet. 2011 Jul 7;7(7):e1002114. doi: 10.1371/journal.pgen.1002114 (PMC3131273; doi:10.1371/journal.pgen.1002114)

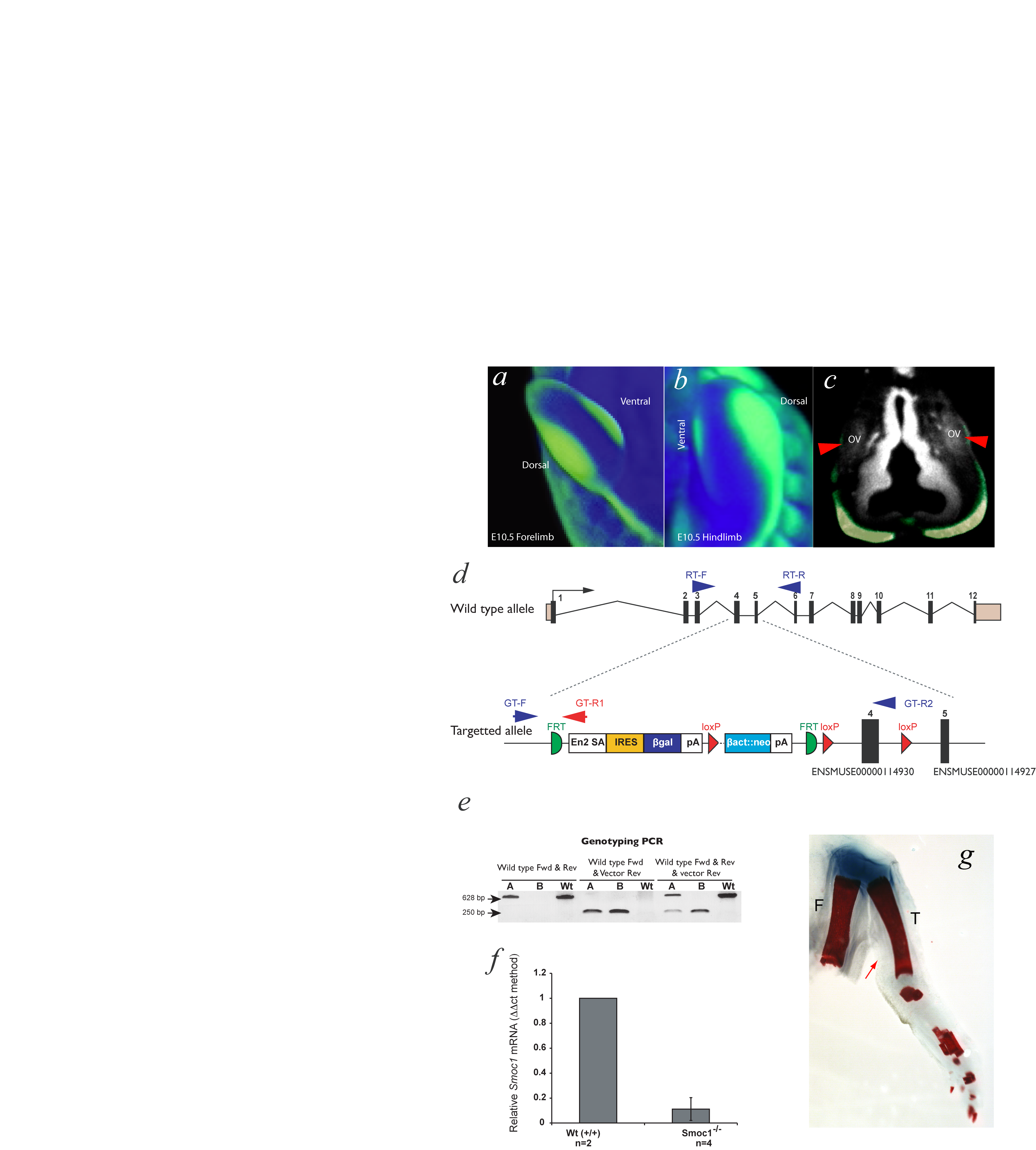

Supplement: Figure S1 — Expression of Smoc1 in the limb buds and eye, and phenotype in Smoc1tm1a/tm1a limbs. (a–c) Magnified images and digital section of the same OPT reconstruction of a wild-type E10.5 embryo shown in Figure 2 with green staining representing Smoc1 expression. (a) Smoc1 expression is seen in both the dorsal and ventral surface of the forelimb buds. (b) In the hindlimb expression is predominantly dorsal and with small region of ventral expression. (c) Digital section showing symmetrical low-level expression of Smoc1 in the surface ectoderm overlying the optic vesicles (OV). (d) Diagram illustrating the gene targeting for the EUCOMM pre-conditional Smoc1 knockout mouse at the genomic level (top) with the relative positions of RT-PCR primers indicated. Targeting cassette (bottom) is specific for exon 4 of Smoc1. Positions of genotyping PCR primers are indicated (GT-F & -R2, blue, are endogenous-gene specific, GT-R1 is cassette-specific) Note that the targeting cassette has the β-galactosidase ORF inserted downstream of an EN2 splice acceptor site (EN2 SA) and internal ribosomal entry site (IRES), allowing for reporter expression in successfully Smoc1-targeted animals (pA is Poly(A) tail). The cassette also features FRT and Cre-recombinase (loxP) sequences for future gene manipulation. Ensembl exon identifiers are given. A table of the phenotypes associated with the targeted allele is available in Table 2 of the main manuscript. (e) Agarose gel indicating each allele identified by genotyping PCR primers. A = phenotypically wild type animal, B = littermate with hindlimb oligodactyly, Wt = wild type unrelated mouse DNA sample. Primer pair 1 (GT-F & GT-R2) amplified wild type allele only, Primer pair 2 (GT-F & GT-R1) amplified the targeted allele only, multiplex reaction (GT-F & GT-R2 & GT-R1) identified both WT and mutant alleles and revealed animal B as a Smoc1 homozygote (Smoc1tm1a/tm1a) and A as a heterozygote (Smoc1+/tm1a). Multiplex reactions were then used as standard fo [file pgen.1002114.s001.tif]

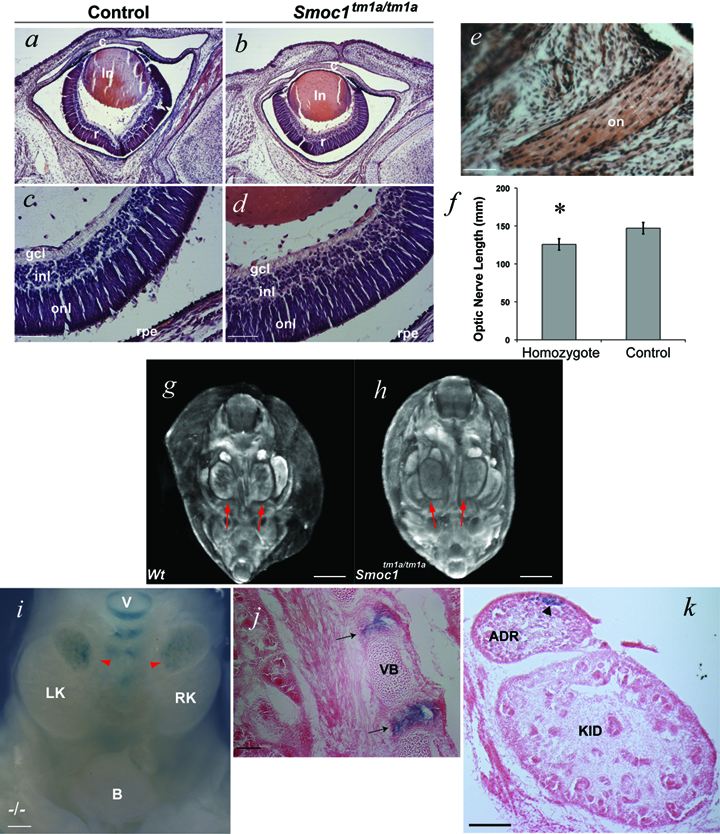

Supplement: Figure S2 — Phenotypic analysis and X-gal staining of Smoc1 mutant animals. (a & b) Low power magnification of H&E stained eyes revealed that mutant eyes were grossly normal but that overall eye size was reduced. (c & d) Higher magnification analysis revealed normal organization and retinal cell-layer lamination in Smoc1tm1a/tm1a mutant eyes. Abbreviations: c, cornea; ln, lens; r, retina; gcl, ganglion cell layer; inl, inner nuclear layer; onl, outer nuclear layer; rpe, retinal pigmented epithelium (Scale bars: a & b = 250 µm; c, d & e = 100 µm). (e) Maximum width measurements were taken for optic nerves (on) of Smoc1tm1a/tm1a mutants (n = 17) and controls (n = 11) from paraffin-embedded sections (f) This plot shows the significantly smaller mean diameter of the optic nerve in mutants compared to control animals with the error bars representing the non-overlapping of 95% confidence limits; 125.7 µm (118.2–133.3) and 147.0 µm (139.6–154.5) respectively. (g,h) Transverse digital sections from OPT scans of E14.5 WT and Smoc1tm1a/tm1a embryos showing an apparently normal size and shape of developing kidneys (arrows). Scale bar = 1 mm. (i) Whole mount X-gal stained Smoc1tm1a/tm1a embryo at E14.5. This embryo was dissected to reveal the developing kidneys. No X-gal positive cells can be seen in the kidneys (LK, left kidney; RK, right kidney) or bladder (B). Stain is clearly seen in both adrenal glands (arrowheads) and in tissue adjacent to the developing vertebrae (V). Scale bar = 500 µm. (j) Sagittal cryosection of 25 µm thickness counterstained with eosin showing strong X-gal staining in the tissue between the developing vertebral bodies (VB). (k) E14.5 stage left kidney seen in i. No stain positive cells can be seen within the kidney (KID) but a cluster of positively staining cells are seen within the adrenal (ADR). Scale bar = 100 µm. (TIF) [file pgen.1002114.s002.tif]
