# Supplementary material for: Loss of the BMP Antagonist, SMOC-1, Causes Ophthalmo-Acromelic (Waardenburg Anophthalmia) Syndrome in Humans and Mice
Source: PLoS Genet. 2011 Jul 7;7(7):e1002114. doi: 10.1371/journal.pgen.1002114 (PMC3131273; doi:10.1371/journal.pgen.1002114)
Supplement: Figure S3 — Alignment of human SMOC-1 Thyroglobulin type-1 (Tg1) domains with Tg1 domains from mouse Smoc-1, Xenopus tropicalis Smoc-1 and Drosophila melanogaster Pentagone. Alignment of the Tg1-1 and Tg1-2 domains from mouse Smoc-1 and human SMOC-1 with the Tg1-2 domains from Xenopus tropicalis Smoc-1 and Drosophila melanogaster Pentagone. The position of identical amino acid residues across all sequences is given by pink shading. The gray shading indicates the conservation of the positions in the Tg1-2 affected by the missense mutations and the nature and position of the mutations is shown in red text below. Key: Tg1 = Thyroglobulin type-1 domain; Tg1-1 = first Tg1 in the peptide; Tg1-2 = second Tg1 in the peptide; hSMOC1 = human SMOC1; mSmoc1 = mouse Smoc1; xtSmoc1 = Xenopus tropicalis Smoc1; dmPent = Drospophila melanogaster Pentagone protein; Q9h4F8 etc are UniProt accession numbers. (DOC) [file pgen.1002114.s003.doc]

92 QSKCRLERAQALEQAK-KPQEAVFVPECGEDGSFTQVQCHTYTG--YCWCVTPD-GKPISGSSVQNKTPVC 158 Q9H4F8 hSMOC1_Tg1-1

91 QSKCRLERAQALEQAK-KPQEAVFVPECGEDGSFTQVQCHTYTG--YCWCVTPD-GKPISGSSVQNKTPVC 157 Q8BLY1 mSmoc1_Tg1-1

224 VYSCDQERQSALEEAQQNPREGIVIPECAPGGLYKPVQCHQSTG--YCWCVLVDTGRPLPGTSTRYVMPSC 292 Q9H4F8 hSMOC1_Tg1-2

234 VHSCDQERQSALEEARQNPREGIVIPECAPGGLYKPVQCHQSTG--YCWCVLVDTGRPLPGTSTRYVMPSC 302 Q8BLY1 mSmoc1_Tg1-2

234 --SCDQERQSALEEAKLNPREGIVIPECAPGGLYKPVQCHQSTG--YCWCVLVDTGRPLPGTSTRYETPVC 302 B8PXJ7 xtSmoc1_Tg1-2

303 ---CWMDQSVTLEEQGHGGKSVLFVPQCLPDGRYQRIQCYSSTSTSYCWCVNEDTGKSIPGTSVKNKRPQC 370 Q86MK1 dmPent_Tg1-2

* :: :**: :. :.:*:* .* : :**: *. ***** * *:.:.*:*.: * *

C N OAS missense mutations

278 283 Position in hSMOC1
